# MeCP2 Deficiency in Neuroglia: New Progress in the Pathogenesis of Rett Syndrome

**DOI:** 10.3389/fnmol.2017.00316

**Published:** 2017-10-04

**Authors:** Xu-Rui Jin, Xing-Shu Chen, Lan Xiao

**Affiliations:** ^1^Department of Histology and Embryology, Faculty of Basic Medicine, Collaborative Program for Brain Research, Third Military Medical University, Chongqing, China; ^2^The Cadet Brigade of Clinic Medicine, Third Military Medical University, Chongqing, China

**Keywords:** Rett syndrome (RTT), MeCP2, astrocyte, oligodendrocyte, microglia

## Abstract

Rett syndrome (RTT) is an X-linked neurodevelopmental disease predominantly caused by mutations of the methyl-CpG-binding protein 2 (MeCP2) gene. Generally, RTT has been attributed to neuron-centric dysfunction. However, increasing evidence has shown that glial abnormalities are also involved in the pathogenesis of RTT. Mice that are MeCP2-null specifically in glial cells showed similar behavioral and/or neuronal abnormalities as those found in MeCP2-null mice, a mouse model of RTT. MeCP2 deficiency in astrocytes impacts the expression of glial intermediate filament proteins such as fibrillary acidic protein (GFAP) and S100 and induces neuron toxicity by disturbing glutamate metabolism or enhancing microtubule instability. MeCP2 deficiency in oligodendrocytes (OLs) results in down-regulation of myelin gene expression and impacts myelination. While MeCP2-deficient microglia cells fail in response to environmental stimuli, release excessive glutamate, and aggravate impairment of the neuronal circuit. In this review, we mainly focus on the progress in determining the role of MeCP2 in glial cells involved in RTT, which may provide further insight into a therapeutic intervention for RTT.

## Introduction

Rett Syndrome (RTT) is an X-linked autism spectrum disorder that affects 1 in every 10,000–15,000 newborns in the United States (Chahrour and Zoghbi, 2007). It is specially characterized by a period of seemingly normal development that lasts for 6–18 months after birth. Subsequently, microcephaly and stereotypic hand wringing start to appear (Nomura, 2005) and more special symptoms appear as the age increases, such as a loss of motor coordination, ataxia, gait apraxia, seizures, poor sleep efficiency or parkinsonian features (Roze et al., 2007). In an overwhelming majority (more than 95%) of RTT patients, the syndrome is caused by mutations in a gene called methyl-CpG-binding protein 2 (MeCP2), a transcriptional corepressor that can bind to methylated CpG islands and complex with Sin3 homolog A (Sin3A) and histone deacetylases (HDACs) to regulate gene expression (Lyst and Bird, 2015). Moreover, different mutations are associated with disease severity, and there are approximately 30 types of mutations that can cause RTT. Patients with the R270X mutation and frame shift deletions in a (CCACC)_n_-rich region present with the most typical symptoms (Bienvenu et al., 2000).

Generally, the symptoms of RTT are attributed to neuronal dysfunction. Substantial evidence shows that neuronal, morphological and functional abnormalities are involved in RTT pathogenesis. For instance, MeCP2-deficient neurons have an abnormal morphology (e.g., fewer dendritic spines and reduced arborization) (Zhou et al., 2006; Smrt et al., 2007; Palmer et al., 2008). The number of synapses is decreased in hippocampi of MeCP2-null mice and conversely, the change was elevated in MeCP2-overexpressing mice (Johnston et al., 2001; Chao et al., 2007; Banerjee et al., 2012). Moreover, the re-expression of *MECP2* in MeCP2-null neurons effectively rescued behavioral abnormalities in mice (Luikenhuis et al., 2004).

However, increasing evidence has shown that white matter damage and/or glial cell (i.e., astrocyte, oligodendrocyte and microglia) dysfunction induced by a change in the DNA methylation state is also involved in the pathogenesis of RTT (Ballas et al., 2009; Maezawa and Jin, 2010; Okabe et al., 2012; Durand et al., 2013; Nguyen et al., 2013). Recently, it was reported that MeCP2-null astrocytes are incapable of supporting the normal development of co-cultured wild-type (WT) neurons (Williams et al., 2014). MeCP2-null microglia and astrocytes have been reported to be toxic to neurons through non-cell autonomous mechanisms, including a slower rate of glutamate (Glu) clearance and release of excessive Glu, as well as glial connexin (Maezawa et al., 2009; Maezawa and Jin, 2010). This latter finding proposes a viewpoint that RTT is not simply a disease of neurons alone, but a complex disease in which glial cells might play a vital role in the pathological process. Hence, we will attempt to summarize the progress of glial abnormalities involved in the pathogenesis of RTT in this review.

## MeCP2 and RTT

**FIGURE 1:**
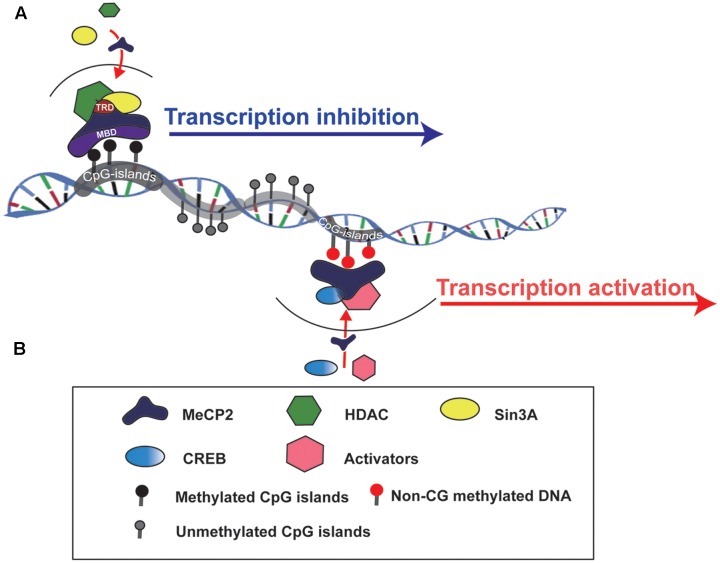
Schematic to show how MeCP2 regulates target gene expression. **(A)** MeCP2 recruits a transcriptional corepressor complex containing Sin3A and histone deacetylase (HDAC) to methylated CpG islands and results in target gene transcription inhibition. TRD, transcriptional repression domain; MBD, methyl-CpG-binding domain. **(B)** MeCP2 is able to active gene transcription by recruiting CREB and other transcriptional factors to non-CG methylted DNA regions.

MeCP2 is one of the members of the methyl-CpG-binding domain protein (MBD) family, which is functionally involved in chromatin remodeling or transcriptional regulation. There are two crucial domains in MeCP2:, one is MBD and another is the transcriptional repression domain (TRD), which can recruit different protein partners, such as HDACs and Sin3A, to form a transcriptional repression complex and regulate target gene expression (Du et al., 2015) (**Figure 1**). The *MeECP2* gene consists of four exons (exon 1–4) and three introns (intron 1–3) and is located on the X chromosome. The transcriptional level of MeCP2 exon 1 (E1) is much higher than other exons in the brain, and mutations in MeCP2 E1 are sufficient to cause RTT (Fichou et al., 2009). Furthermore, the MeCP2 isoform has a time-specific expression pattern during brain development. MeCP2 E1 in the mouse hippocampus was detected as early as at E14, whereas MeCP2 E2 was detected at E18 (Olson et al., 2014). Generally, *MECP2* was believed to bind to methylated CpG islands; however, a recent study showed that MeCP2 can bind to non-CG methylated DNA and influence the transcription of disease-relevant genes in the adult mouse brain (Chen et al., 2015; Luo and Ecker, 2015) (**Figure 1**). Those results provide insight into the molecular mechanism of MECP2 in the delayed onset of RTT.

Generally, almost 95% of RTT patients carry mutations in the MeCP2 gene, and recent findings demonstrated that two additional genes, cyclin-dependent kinase like 5 (CDKL5) (Evans et al., 2005) and fork head box G1 (FOXG1), can also be involved in the pathogenesis of this syndrome (Mencarelli et al., 2010). Furthermore, CDKL5 has been shown to have the ability to promote the release of MeCP2 from DNA by phosphorylating MeCP2 (Mari et al., 2005; Bertani et al., 2006), while a direct functional relationship between these two molecules in RTT is controversial.

## MeCP2 Deficiency and Neuronal Dysfunction

The function of MeCP2 in brain is multifarious, including modulation of neurogenesis, synaptic development and maintenance of neural circuits (Chahrour and Zoghbi, 2007; Banerjee et al., 2012; Lyst and Bird, 2015). It was demonstrated that MeCP2 is essential for neurogenesis in Xenopus embryos, and deficiency of MeCP2 resulted in a decreased number of neuronal precursors (Stancheva et al., 2003). *In vitro*, MeCP2 mutant mesenchymal stem cells presented impaired neural differentiation and increased the rate of senescence (Squillaro et al., 2012). MeCP2-deficient neurons have decreased numbers of axons and dendrites (Nguyen et al., 2012), and the neurite complexity was reduced in cultured MeCP2-null embryonic primary cortical neurons (Vacca et al., 2016), indicating that MeCP2 plays a crucial role in modulating neuronal differentiation and terminal maturation. Recent studies have revealed that MeCP2 is also involved in neuronal cell fate specification and migration (Feldman et al., 2016). It was found that neural progenitor cells (NPCs) lacking MeCP2 exhibited delayed corticogenesis with respect to abnormal migration of NPCs from the subventricular and ventricular zones into the cortical plate (Bedogni et al., 2016).

Some of the abnormal social behaviors of patients with RTT, such as anxiety and autistic features, are thought to be caused by MeCP2 deficiency in the neurons of certain special brain areas, including the forebrain, hypothalamus and basolateral amygdala (Armstrong et al., 1998; Gemelli et al., 2006; Fyffe et al., 2008; Chao et al., 2010; Williams et al., 2014). *MECP2* conditional knockout in glutamatergic neurons, but not in inhibitory neurons, leads to more serious RTT-like symptoms in mice (Meng et al., 2016). It has been shown that the balance between Glu excitatory synapses and GABAergic (gamma-amino butyric acid) inhibitory synapses is disrupted in RTT (Nelson and Valakh, 2015). MeCP2 knockdown reduces the excitatory synapse number and attenuates synaptic scale-up by reducing the expression of metabotropic glutamate receptor 2 (GluR2), and there is a major down-regulation of GABAergic inhibitory synapses in MeCP2 knockout mice (He et al., 2014; Kang et al., 2014). Thus, correcting the abnormal MeCP2 level by adding the gene back or over-expressing is a valid method to study the function of MeCP2 and treatment for RTT. For instance, dendritic abnormalities and behavioral changes can be ameliorated by reactivation of MeCP2 expression in MeCP2-null mice (Armstrong et al., 1998; Stearns et al., 2007; Robinson et al., 2012). Recently, MECP2 gene therapy by intracisternal injection of transgenic adeno-associated virus 9 (AAV9/hMECP2) has been shown to extend survival of MeCP2-deficient (Mecp^2-/y^) mice without apparent toxicity (Matagne et al., 2017; Sinnett et al., 2017). Moreover, as RTT is always caused by heterozygous mutations in an X-linked gene and there exists a prevalent wild-type MeCP2 allele on the inactive X chromosome, reactivation of the inactive X chromosome-linked wild-type allele may represent another way to rescue MeCP2 deficiency. Recently, several trans-acting X-chromosome inactivation factors (XCIFs) have been identified and the inhibitor of two XCIFs (PDPK1, AURKA) has been demonstrated to exhibit reactive expression of the WT MeCP2 allele on the inactive X chromosome, which can be considered as a potential therapy for RTT (Bhatnagar et al., 2014).

In addition to RTT, evidence has shown that MeCP2 regulates the expression of its downstream genes, such as brain-derived neurotrophic factor (BDNF) (Chen et al., 2003) and ubiquitin-protein ligase E3A (UBE3a), the latter being involved in Angelman syndrome (Makedonski et al., 2005). In addition, MeCP2 also regulates the expression of distal-less homeobox 5/6 (Dlx5/6) genes, which are necessary for spinal skeletal development and are related to definite symptoms of RTT patients, such as scoliosis and microcephaly (Nakashima et al., 2010).

## MeCP2 Deficiency and Glial Cell Dysfunction

Glial cells are non-excitable cells that are functional support neurons and maintain the stability of neuronal structure and function. In the central nervous system (CNS), glial cells mainly include astrocytes, OLs and microglia. Previous studies have revealed that MeCP2 is present in a majority of neurons but is absent from glial cells (Shahbazian et al., 2002). However, increasing evidence suggests that the abnormality of MeCP2 also plays an important role in white matter damage and/or glial dysfunction in RTT (Ballas et al., 2009; Maezawa and Jin, 2010; Okabe et al., 2012; Durand et al., 2013; Nguyen et al., 2013). Mice with MeCP2 loss specifically in glial cells presented Rett-like symptoms due to neuronal toxicity via a non-cell autonomous mechanism, and the restoration of MeCP2 in glial cells can rescue some of these defects (Lioy et al., 2011; Nguyen et al., 2013; Cronk et al., 2015). Evidence from magnetic resonance spectroscopy (MRS) indicated an increased glia-to-neuron ratio in the white matter of RTT patients along with ongoing axonal damage and glial abnormalities (Khong et al., 2002). The contribution of MeCP2 deficiency to dysfunction of different types of glial cells varies in RTT pathogenesis.

## MeCP2 in Astrocytes

Astrocytes are the most abundant of all glial cell types and are well known for supporting neurons and maintaining brain function. Although the expression level of MeCP2 in astrocytes is lower, it is important for astrocyte differentiation and function. After *MECP2* was specifically knocked out in neural stem cells (NSCs), these NSCs tend to differentiate into more astrocytes (Andoh-Noda et al., 2015). While the growth rate of MeCP2-deficient astrocytes is significantly slower and more interleukin (IL)-1β and IL-6 are released in response to immune stimulation *in vitro*, no obvious morphological change was found in those cells (De Filippis et al., 2012). MeCP2-deficient astrocytes that were differentiated from induced pluripotent stem cell (iPSC) lines from RTT patients have negative regulatory effects on neuronal morphology and function (Williams et al., 2014). Moreover, subsequent studies found that MeCP2-null astrocytes have certain abnormalities in target gene regulation and are toxic to neurons mainly due to abnormal Glu metabolism. Similarly, it was shown that a state of MeCP2 deficiency can spread in the brain of MeCP2^-/+^ mice via a non-cell autonomous mechanism (Maezawa et al., 2009). This suggests that abnormal astrocytes are able to deteriorate the function of neurons, which speeds up the process of RTT. It was found that preferential re-expression of Mecp2 in astrocytes dramatically improved RTT symptoms and the lifetimes of MeCP2-deficient mice (Kifayathullah et al., 2010; Lioy et al., 2011; Zachariah et al., 2012).

### Target Gene Dysregulation in MeCP2-Null Astrocytes

The expression of astroglial marker genes GFAP and S100β was significantly higher in MeCP2-null astrocytes than that in WT astrocytes (Forbes-Lorman et al., 2014). It was noted that *MECP2* small interference RNA (siRNA) increased the expression of GFAP in the female amygdala (Forbes-Lorman et al., 2014). These observations suggest that GFAP and S100β genes are suppressed by MeCP2. Although the mechanism by which MeCP2 regulates these two genes is unclear, evidence has shown that MeCP2 E1 can couple with the Sin3A/HDAC complex, which can bind to the GFAP promoter and regulate GFAP transcription. On embryonic day 11.5 (E11.5), the promoter of the GFAP gene is highly methylated, which may facilitate assembly of MeCP2-Sin3A/HDAC complexes and suppress GFAP gene transcription. On E14, the promoter of the GFAP gene is demethylated, which may lead to the disintegration of this complex and results in GFAP gene expression. In the early stages of astrocyte differentiation, MeCP2 also has a vital effect by binding to the promoter of the S100β gene, while this binding is gradually reduced in the later stages, as demethylation of a specific CpG site occurs (Cheng et al., 2011; Forbes-Lorman et al., 2014).

In addition to astroglial marker genes, many other target genes such as solute carrier family member 38, member 1 (Slc38a1), neuronal regeneration-related protein (Nrep), and nuclear receptor subfamily 2, group F, member 2 (Nr2f2) are also regulated by MeCP2. Slc38a1 is a rate-limiting transporter of glutamine (Gln) across the plasma membrane, Nrep is a transcriptional factor that is involved in glial mobility and neoplasia, and Nr2f2 is a transcription factor that is necessary for glial differentiation from NSCs (Mackenzie and Erickson, 2004; Yasui et al., 2013; Delepine et al., 2015). In addition, Nr2f2 can regulate some target genes including those encoding chromogranin B (Chgb), chemokine (C-C motif) ligand 2 (CCL2), and lipocalin 2 (LCN2). Chgb is a highly efficient system that is directly involved in monoamine accumulation. In MeCP2-deficient mice, the expression of Nr2f2 is up-regulated, which may down-regulate Chgb and cause excess monoamine accumulation in the extracellular fluid and impair neurons. This finding suggests that influence of MeCP2 deficiency can be aggravated through a downstream target gene cascade in astrocytes (Kloukina-Pantazidou et al., 2013; Delepine et al., 2015). Interestingly, in response to LPS stimulation, MeCP2-deficient astrocytes released fewer cytokines such as IL-1β and IL-6 (Maezawa et al., 2009).

### Microtubule Instability in MeCP2-Null Astrocytes

Cellular morphology, division, migration and intracellular transportation of vesicles are controlled by microtubules (MTs), which assemble from α- and β-tubulin dimers. The acetylation modification of tubulin is deemed to be a characteristic of stable MTs (Palazzo et al., 2003). In *Mecp2*^308/y^ and *Mecp2* p. Arg294^∗^ iPSC-derived astrocytes, the level of acetylated tubulin is reduced, and histone deacetylase 6 (HDAC6) is overexpressed (Delepine et al., 2016). A similar result was also reported in MeCP2-deficient fibroblasts and MeCP2-null neurons (Gold et al., 2015). MT growth speed of Mecp2^308/y^ astrocytes was higher, and MT polymerization was significantly increased. Moreover, MT-dependent lysosome vesicle mobility was obviously increased in both *Mecp2*^308/y^ and *Mecp2* p.Arg294^∗^ iPSC-derived astrocytes; however, the percentage of highly directional vesicles was reduced (Delepine et al., 2016). A special type of directional vesicle cellular transport can regulate Glu uptake, which suggests an abnormal Glu uptake rate may be associated with MT-dependent vesicle mobility in RTT (Li et al., 2015). The aforementioned phenomenon was related to the decreased expression of stathmin 2 (STMN2), which could inhibit MT polymerization (Nectoux et al., 2012). Moreover, expression of TUBA1B, which encodes the ubiquitous α-tubulin, is down-regulated in brain tissue of patients with RTT (Abuhatzira et al., 2009). These findings suggest a role of MeCP2 as an activator for MT-associated genes. Molecularly, a recent study revealed a potential mechanism by which sumoylated MeCP2 releases CREB from the repressor complex and increases the transcription of CREB-regulated genes such as BDNF (Tai et al., 2016). Interestingly, sumoylation of MeCP2 was found to be decreased in RTT, and further study is warranted to examine the sumoylation status of MeCP2 in glial cells of RTT. In addition, epothilone D, a brain-penetrating MT-stabilizing natural product, can rescue MT growth velocity, and epothilone D corrects the abnormal behavioral symptoms of *Mecp2*^308/y^ mice (Delepine et al., 2016). Treatment via targeting MTs seems to be a new approach for RTT therapy. Notably, when co-cultured with MeCP2-deficient fibroblasts, MT stability of WT human fibroblasts is reduced (Delepine et al., 2013). All of this evidence suggests that MT impairment seems to be a common phenomenon of MeCP2 deficiency.

### Abnormal Glutamate Metabolism in MeCP2-Deficient Astrocytes

**FIGURE 2:**
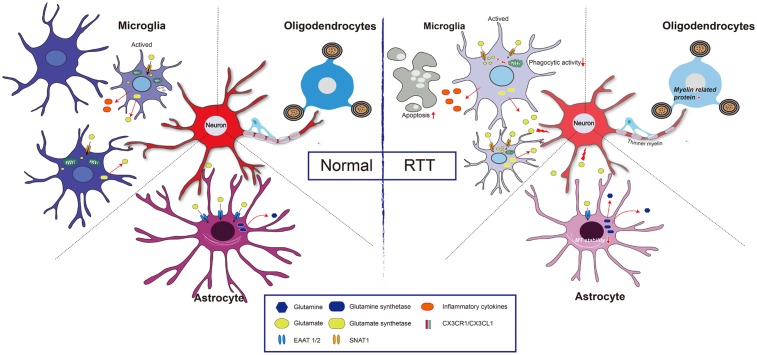
Glia and neuron interaction under pathologic condition of RTT. Normal: Extracellular glutamate (Glu) can be uptake by excitatory amino acid transporter 1 and 2 (EAAT1/2) in astrocyte and sodium-coupled neutral amino acid transporter 1 (SNAT1) in microglial cell, and is utilized by glutamine synthetase to reduce its toxicity to neuron. Both astrocytes and microglia can be active upon stimulation, and the active microglia has capacity of phagocytosis. Furthermore, CX3CR1 (a chemokine receptor in microglial cell) and its neuronal ligand CX3CL1 mediates neuron-microglia interaction. Myelinating oligodendrocyte is important to maintain axonal integrity. RTT: MeCP2 deficiency in astrocytes leads to a decreased expression of EAAT1/2, and increased expression of glutamine synthetase, which results in a high concentration of extracellular Glu, and thus impairs neuronal activity. Additionally, those astrocytes present decreases microtubule stability. In microglia, MeCP2 deficiency results in decreased phagocytic activity and overloads of Glu due to upregulation of SNAT1. The MeCP2-deficient in oligodendrocyte results in thinner myelin due to down-regulation of myelin-related genes.

Glutamate is an important signaling molecule in the CNS. At a high extracellular concentration, it is a potent cytotoxin that can induce both neuronal and glial death through excitotoxicity or oxidative stress. Extracellular glutamate concentration is maintained at an appropriate level predominantly by active transport mediated by excitatory amino acid transporters (EAATs) of astrocytes (Lehmann et al., 2009). Normally, when astrocytes are incubated with high levels of extracellular Glu, EAAT1/EAAT2 expression is rapidly decreased. However, when the down-regulation of EAAT1/2 was impaired, EAAT1/EAAT2 expression had no obvious changes in MeCP2-null astrocytes (Okabe et al., 2012). Regarding the underlying mechanism, it was found that HDAC I and II served as repressors for EAAT2 promoter activity (Karki et al., 2014), thus, MeCP2 deficiency may fail to recruit HDACs to inhibit EAAT gene expression upon environment stimulation. Notably, the Glu clearance rate of MeCP2-null astrocytes was lower than that of WT astrocytes *in vitro* (Okabe et al., 2012). Moreover, the glutamine synthetase (GS) protein was significantly higher in MeCP2-null astrocytes than that in WT astrocytes (Okabe et al., 2012). All of this evidence seems to support the concept that both Glu clearance and production are abnormal in MeCP2-deficient astrocytes, and that these abnormalities may contribute to the pathological process of RTT (**Figure 2**).

### Spread of MeCP2 Deficiency in Astrocytes

An interesting phenomenon is that MeCP2 expression in *Mecp2*^-/+^ mice is much lower at 7 months than that at 1 month, when proliferation rates of astrocytes are higher. *In vitro*, MeCP2 expression was significantly decreased in WT astrocytes that were co-cultured with MeCP2-deficient astrocytes for a long period, indicating that MeCP2 deficiency can spread among astrocytes progressively and eventually lead to a dysfunctional brain (Maezawa et al., 2009). Recent studies have shown that MeCP2 deficiency can spread among astrocytes via connexin, especially connexin 43 (Cx43)-mediated gap junction. When Cx43 was knocked down with siRNA, the spread of MeCP2 deficiency was significantly reduced (Maezawa et al., 2009). This finding suggests that astroglial Cx may be a new target for preventing the phenotype of RTT.

### Abnormal Astrocytes May Be the Main Contributor to the Disordered Breathing Pattern

RTT symptoms always include a severely disordered breathing pattern and reduced CO_2_ sensitivity. In the respiratory control center, highly chemosensitive astrocytes can respond to the physiological decrease in pH with a vigorous increase in intracellular Ca^2+^ and release of ATP (Gourine et al., 2010). MeCP2 deficiency in astrocytes induced an obvious depression of the ventilatory responses to an increased level of CO_2_, which is similar to global MeCP2-null mice (Zhang et al., 2011). Additionally, although neurons constitute the respiratory control network and determine the ventilatory response to CO_2_, MeCP2 mutation selectively in neurons leads to an absolute lower depression of the CO_2_ response than MeCP2 loss in astrocytes (Fyffe et al., 2008; Garg et al., 2015). After re-expression of MeCP2 in astrocytes, the respiratory phenotype is rescued (Garg et al., 2015), suggesting that the disordered breathing pattern may be caused by abnormal astrocytes in RTT patients. However, recent evidence suggests that RTT-like breathing abnormality was only developed in 20% of *Mecp2*^+/-^ mice (Johnson et al., 2015). Imaging of calcium signaling in ventral medullary astrocytes reveals that these phenomena may be related to a reduction in the ability of MeCP2-deficient astrocytes to sense *P*_CO2_/[H^+^] and that the CO_2_-induced [Ca^2+^]_i_ response is impaired. Additionally, the ATP response of MeCP2-deficient astrocytes is not affected. Normally, ATP propagates astrocytic Ca^2+^ excitation, depolarizes chemoreceptor neurons, and induces adaptive increases in breathing. This evidence suggests that the most apparent deficiency of the MeCP2-null astrocyte is the ability to sense physiological increases in CO_2_, while the downstream signaling pathway can still be activated by ATP (Bissonnette and Knopp, 2008; Zhang et al., 2011; Turovsky et al., 2015).

### MeCP2 in Microglia

#### Mecp2 Regulates Microglial Activation

Microglia are considered to be resident myeloid-derived cells distributed throughout the brain and account for over 10% of CNS cells (Kawabori and Yenari, 2015). Microglial activation can occur in response to stimuli such as glucocorticoids, hypoxia, and inflammation (Kiernan et al., 2016). In Mecp2-null mice, microglial cells are relatively small but become larger in response to environmental stimuli compared to those of wild-type mice, and these microglial cells are lost with disease progression. Moreover, the expression of glucocorticoid-induced transcriptional signature genes and a subset of hypoxia-inducible genes were up-regulated in Mecp2-deficient microglia, suggesting the regulatory role of MeCP2 in microglia activation (Cronk et al., 2015) (**Figure 2**). From another point of view, a gene array study confirmed that the function of differentially expressed genes, as MeCP2 deficiency is strongly related to regulating the activation states of microglia (Zhao et al., 2017). In addition, studies have shown that bone marrow transplantation increases the number of microglia in the brain (Derecki et al., 2012). MeCP2-null mice that were transplanted with WT bone marrow partly restored behavior and functional abnormalities (Derecki et al., 2012). However, another study reached the opposite conclusion that WT marrow transplantation did not positively influence MeCP2-null mice (Wang et al., 2015). The beneficial effect declined when the phagocytic activity of these transplanted cells was inhibited and the ability to clear apoptotic targets was reduced. This result indicated that the phagocytic activity of microglia is impaired in MeCP2-null microglia and that a non-cell autonomous mechanism is involved in spreading the MeCP2-deficient state (Maezawa and Jin, 2010). In contrast to microglia, the MeCP2 knockout of myeloid-derived cells led to the release of more tumor necrosis factor-α (TNF-α) and inflammatory cytokines, such as IL-6, TNF-α, and IL-3 (O’Driscoll et al., 2015).

#### Mutant MECP2 in Microglia Impairs the Neuronal Circuit

In the developing CNS, microglia have been demonstrated to play an important role in shaping neuronal circuit structure by microglia-synapse interactions or removing excess synapses, and an etiology of disrupted synaptic function can be detected in mouse models of RTT (Johnston et al., 2001; Noutel et al., 2011). Recent research offered a new viewpoint of the effect of MECP2-defective microglia on the neuronal circuit. It was reported that in MECP2-null mice, microglia contribute to RTT pathogenesis by excessively engulfing and thereby eliminating, which is concomitant with synaptic loss at the end stages of the disease (Schafer et al., 2016). Intriguingly, gain or loss of Mecp2 expression specifically in microglia cannot induce the abovementioned phenomena (Schafer et al., 2016), indicating that abnormalities in microglia may accelerate the pathological process by impairing the neuronal circuit as a secondary effect, not the primary cause, of RTT. In addition, the result of another research study also implicitly demonstrated this point that restoring the expression of Mecp2 in myeloid cells by bone marrow transplantation made no sense in the Mecp2-null mouse (Wang et al., 2015).

A recent study showed that ablating CX3CR1, a chemokine receptor of microglia mediating neuron–microglia interaction by pairing with its neuronal ligand CX3CL1, can rescue the negative effect of *MeCP2*-deficient microglia on neurons. After blocking the interaction, the survival of MECP2-null mice was remarkably improved and behavioral abnormalities were partly restored. These results indicated that blocking intercellular interaction in brains of RTT patients might be a novel therapeutic approach for RTT (Horiuchi et al., 2016).

#### MeCP2-Deficient Microglia Leads to Neurotoxicity

Activation of glutamate receptors and the expression level of Glu transporters are important for maintaining the plasticity of glutamatergic synapses (Groc et al., 2006; Yuan and Bellone, 2013). MeCP2-deficient microglia released a fivefold higher level of Glu, which was associated with neurotoxic or synaptotoxic activity, as the neuronal abnormality can be partially restored by blocking the NMDA receptor (Maezawa and Jin, 2010). In addition,which can release Glu, was significantly up-regulated in MeCP2-null microglia, and the blockade of Cx32 can partially ameliorate the excessive release of Glu (Maezawa and Jin, 2010).

From the perspective of bioenergetics, MeCP2 loss in microglia led to mitochondrial dysfunction by impairing Glu homeostasis (Jin et al., 2015). In MeCP2-deficient microglia, the overexpression of the Glu transporter sodium-coupled neutral amino acid transporter 1 (SNAT1) promoted microglial uptake of Glu; the excessive Glu was translated into the mitochondria, coupled with the rising mitochondrial reactive oxygen and damaged the mitochondria (Jin et al., 2015). As a consequence, adenosine triphosphate (ATP) production is significantly reduced in MeCP2-deficient microglia, and this ATP reduction leads to a greater potential for the apoptosis of abnormal microglia (Jin et al., 2015) (**Figure 2**).

### MeCP2 in Oligodendroglia

Oligodendrocytes are the myelinating cells in the brain that maintain nerve impulse conduction and provide nutrition for axons (recently reviewed in Bergles and Richardson, 2016). There are several markers that distinguish the oligodendroglial cells at different stages. OL progenitor cells (OPCs) are positive for platelet-derived growth factor receptor α (PDGFR-α) or neural antigen 2 (NG2); immature OLs are O4-positive, and mature OLs express myelin-associated glycoprotein (MAG), proteolipid protein (PLP), CNP and myelin basic protein (MBP) (He and Lu, 2013).

Some studies have found the expression of MeCP2 in oligodendroglial lineage cells (KhorshidAhmad et al., 2016), which may be associated with oxidative damage of white matter that occurred in the early stages of RTT (Durand et al., 2013). In MeCP2-null mice, although the OL morphology is unchanged, the expression of myelin-related genes is partially changed in some specific regions. For example, in the frontal cortex of MeCP2-null mice, no changes in global expression levels of the MBP, MAG and PLP were found, but MAG expression was significantly increased in the corpus callosum, while PLP expression was significantly decreased in the cerebellum (Vora et al., 2010). Furthermore, in *Mecp2*^308/y^-mutant mice, GFAP, Cx43, Cx45, Cx40 and Cx32 were unchanged, but the CNP expression level was decreased (Wu et al., 2012). Additionally, cultured OLs with MeCP2 knocked down exhibited an increase in myelin genes, including MBP, PLP, myelin oligodendrocyte glycoprotein (MOG), myelin-associated oligodendrocyte basic protein (MOBP) and Ying Yang 1 (YY1) (KhorshidAhmad et al., 2016), indicating MeCP2 is a negative regulator for myelin protein expression (Sharma et al., 2015). As a co-repressor of MeCP2, HDACs have also been found to be functionally involved in oligodendroglial development (Huang et al., 2015). Moreover, when MeCP2 was specifically knocked out in NG2^+^ OPCs, the mice displayed more active behaviors, with severe hind limb-clasping phenotypes (Nguyen et al., 2013). The PLP and MBP expression levels were lower in those mice, and thinner myelin was present. After restoring the expression of MeCP2 in the OPCs, the ethological abnormality was significantly corrected in both female and male mice (Nguyen et al., 2013) (**Figure 2**). It is noteworthy that MeCP2 re-expression in OPCs, compared with that in astrocytes or microglia cells, shows more potent ability to prolong the lifespan of MeCP2^stop/y^ mice (Nguyen et al., 2013). However, the expression of myelin protein (MBP) was mildly or not rescued (CNP, MOG, or PLP) after re-expression of MeCP2 in oligodendrocytes in otherwise MeCP2-null mice, suggesting that a likely non-cell autonomous mechanism regulated the expression of OL-related genes (Nguyen et al., 2013). This mechanism is maybe related to the excessive Glu released by other glial cell types, such as astrocytes or microglia, because excessive Glu might partly restore the development of OLs through activating *n*-methyl-D-aspartic acid (NMDA) receptors in MeCP2-null brains (Lundgaard et al., 2013). In addition, white matter damage in RTT patients has been related to impaired nuclear factor kappa-B (NF-κB) signaling: there were additional copies of the inhibitor of the kappa light polypeptide gene, kinase gamma (IKBKG) enhancer in B-cells of patients with Xq28 duplications, and three of the five presented white matter anomalies in brain MRI (Philippe et al., 2013).

### Perspectives

In this review, we summarized glial abnormalities induced by MeCP2 deficiency and how these cells are toxic to neurons and disturb the neural circuit (**Table 1**). The mice with MeCP2-null specifically in glial cells showed behavioral and neural synaptic abnormalities that are similar to those in MeCP2-null mice, and re-expression of MeCP2 in glial cells can restore those abnormalities and prolong the life span of MeCP2-null mice. Additionally, we discussed the role of MeCP2 in glial cells to gain a better understanding of the pathology process of RTT and ultimately a new glial-target strategy for RTT treatment. For instance, Glu metabolism abnormalities may be a predominant component of the RTT pathology process, especially in glial cells. Some drugs such as riluzole, a glutamatergic modulator that has been approved for treating amyotrophic lateral sclerosis (ALS) by primarily rectifying extensive Glu levels, may also be utilized to treat RTT. Moreover, the regulatory mechanism of MeCP2 in the oligodendroglial development involved in RTT pathogenesis is understudied.

**Table 1:** A summary of the abnormal features in glial cells induced by MeCP2 abnormalities.

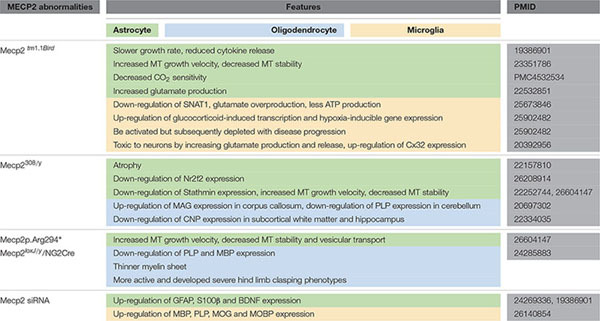

## Author Contributions

X-RJ and X-SC wrote the manuscript, LX designed and revised the manuscript.

## Conflict of Interest Statement

The authors declare that the research was conducted in the absence of any commercial or financial relationships that could be construed as a potential conflict of interest.
